# Probing the vibrational spectroscopy of the deprotonated thymine radical by photodetachment and state-selective autodetachment photoelectron spectroscopy *via* dipole-bound states†
†Electronic supplementary information (ESI) available. See DOI: 10.1039/c5sc00704f



**DOI:** 10.1039/c5sc00704f

**Published:** 2015-03-17

**Authors:** Dao-Ling Huang, Hong-Tao Liu, Chuan-Gang Ning, Guo-Zhu Zhu, Lai-Sheng Wang

**Affiliations:** a Department of Chemistry , Brown University , Providence , Rhode Island 02912 , USA . Email: Lai-Sheng_Wang@brown.edu; b Shanghai Institute of Applied Physics , Chinese Academy of Sciences , Shanghai 201800 , China; c Department of Physics , State Key Laboratory of Low-Dimensional Quantum Physics , Tsinghua University , Beijing 100084 , China

## Abstract

Deprotonated thymine can exist in two different forms, depending on which of its two N sites is deprotonated: N1[T–H]^–^ or N3[T–H]^–^. Here we report a photodetachment study of the N1[T–H]^–^ isomer cooled in a cryogenic ion trap and the observation of an excited dipole-bound state. Eighteen vibrational levels of the dipole-bound state are observed, and its vibrational ground state is found to be 238 ± 5 cm^–1^ below the detachment threshold of N1[T–H]^–^. The electron affinity of the deprotonated thymine radical (N1[T–H]˙) is measured accurately to be 26 322 ± 5 cm^–1^ (3.2635 ± 0.0006 eV). By tuning the detachment laser to the sixteen vibrational levels of the dipole-bound state that are above the detachment threshold, highly non-Franck–Condon resonant-enhanced photoelectron spectra are obtained due to state- and mode-selective vibrational autodetachment. Much richer vibrational information is obtained for the deprotonated thymine radical from the photodetachment and resonant-enhanced photoelectron spectroscopy. Eleven fundamental vibrational frequencies in the low-frequency regime are obtained for the N1[T–H]˙ radical, including the two lowest-frequency internal rotational modes of the methyl group at 70 ± 8 cm^–1^ and 92 ± 5 cm^–1^.

## Abstract 2

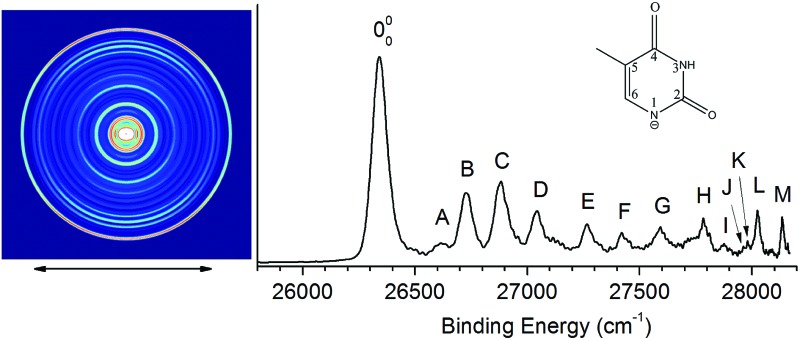
High-resolution state-selective autodetachment photoelectron spectroscopy *via* dipole-bound states and photodetachment spectroscopy of cryogenically cooled deprotonated thymine anions are reported.

## 1. Introduction

Deoxyribonucleic acid (DNA) carries fundamental genetic information in all living organisms. It has been found that radiation damage can cause mutagenic changes to the sequence of nucleobases, leading to miscoded proteins with potentially fatal biological consequences.^1^ To understand the mechanisms of radiation damage, it is critical to investigate the important DNA building blocks, such as nucleobases, at the molecular scale. Thymine, as a unique nucleobase in DNA, has drawn significant attention both theoretically^2–6^ and experimentally.^7–13^ Upon exposure to radiation, thymine undergoes dissociative electron attachment (DEA), which results in a reactive deprotonated thymine species.^14–16^ DEA plays a critical role in the radiation damage of biological systems by breaking chemical bonds through low-energy electrons.^17^ Thus, knowledge about deprotonated thymine radicals ([T–H]˙) and anions ([T–H]^–^) is important in understanding the detailed mechanisms of DEA inside DNA.

**Fig. 1:**
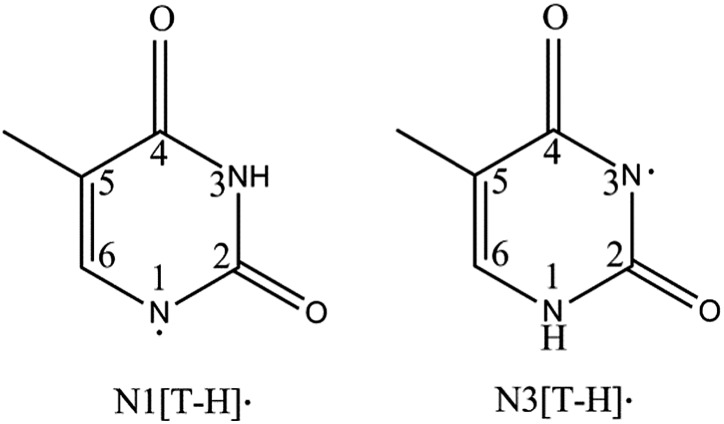
Structures of the two deprotonated thymine radical isomers formed from deprotonation at each N site.

There are two [T–H]˙ isomers formed by N–H bond cleavage from the N1 or N3 site, designated as N1[T–H]˙ and N3[T–H]˙, respectively, as shown in Fig. 1. DEA experiments show that both isomers are produced, depending on the electron kinetic energies.^14^ Theoretical calculations^18–22^ predicted the electron affinities (EAs) of the N1[T–H]˙ and N3[T–H]˙ isomers to be 3.2–3.4 eV and 3.7–4.5 eV, respectively. Isomers formed by C–H bond cleavages are much less stable and have much lower EAs. Due to the short lifetime and complicated isomerization of [T–H]˙, the study of [T–H]˙ has been challenging experimentally. In 2007, a study of [T–H]˙ was reported by Parsons *et al.* using anion photoelectron (PE) imaging at 354.84 nm with limited spectral resolution.^23^ In this study, an EA of 3.250 ± 0.015 eV was obtained for [T–H]˙ and was attributed to the N1[T–H]˙ isomer by comparing the observed PE spectrum with Franck–Condon simulations.

A molecule with a sufficiently large dipole moment was predicted to be able to weakly attract an electron to form a so-called dipole-bound state (DBS),^24–27^ which was observed experimentally.^28–34^ A negative ion can form excited DBSs near the detachment threshold, if the corresponding neutral species of the anion is polar with a large dipole moment. Such excited DBSs are analogous to Rydberg states in neutral molecules, and the extra electron in the dipole-bound anions has little effect on the neutral cores. Excited DBSs were first observed in photodetachment cross sections of organic anions.^35–38^ Because the electron binding energies of the DBSs are low relative to the detachment threshold, ro-vibrational excitations in the DBSs can lead to electron autodetachment *via* vibronic coupling. Rotational autodetachment has been observed *via* DBS, yielding high-resolution spectroscopy for dipole-bound anions.^39–46^ However, due to spectral congestion at room temperature, especially for large and complex anions, only a limited number of such high-resolution detachment spectroscopy experiments have been reported. In particular, autodetachment-enhanced photoelectron spectroscopy (PES) from a DBS has not been reported until recently. In 2013, we reported a DBS for the phenoxide anion cooled in a temperature-controlled ion trap and observed mode-specific vibrational autodetachment from selective vibrational levels of the DBS.^47^ The vibrational frequencies of the DBS were measured to be the same as those of the neutral species, suggesting that high-resolution vibrational spectroscopy of dipolar radicals can be achieved by vibrational autodetachment from the DBSs of cold anions. The Δ*v* = –1 vibrational propensity rule,^48^ which was initially developed for autoionization from Rydberg states,^49^ was observed to be obeyed in the autodetachment from DBS.

Subsequently, we obtained high-resolution vibrational spectroscopy of the deprotonated uracil radical (N1[U–H]˙) *via* autodetachment from vibrational levels of the DBS of cold deprotonated uracil anions.^50^ The observation of a DBS in N1[U–H]^–^ motivates us to revisit thymine, which has a very similar structure to uracil except that a methyl group replaces the H atom in the 5-C site of uracil (Fig. 1). Because of this methyl group, thymine plays a significantly different role to uracil in biological systems, that is, thymine serves as a unique DNA base while uracil is an RNA base. Considering the structural similarity between uracil and thymine, we suspected that an excited DBS should also exist in [T–H]^–^, allowing us to probe the vibrational properties of [T–H]˙. In fact, the thymine molecule has a large dipole moment and can form a dipole-bound anion (T^–^) in the ground electronic state, which was produced previously by Rydberg electron transfers and studied by anion PES.^34,51,52^ The PE spectra yielded a binding energy for the DBS of T^–^ as 60–70 meV with little vibrational structure,^34,52^ confirming that the dipole-bound electron in T^–^ has little effect on the structure of T.

In the current article, we report a photodetachment and high-resolution PE imaging study of deprotonated thymine anions cooled in a cryogenic ion trap. The observed anion is identified as the N1[T–H]^–^ isomer unambiguously with significantly improved spectral resolution. The EA of N1[T–H]˙ is measured accurately to be 26 322 ± 5 cm^–1^ (3.2635 ± 0.0006 eV). More importantly, we have observed an excited DBS for N1[T–H]^–^, 238 ± 5 cm^–1^ below the electron detachment threshold. Using photodetachment spectroscopy, we observe the ground state and seventeen vibrational levels of the DBS. Sixteen high-resolution resonant PE images and spectra are obtained by tuning the detachment laser to the sixteen vibrational levels that are above the detachment threshold. Significantly richer vibrational information is obtained in the resonant PE spectra *via* state- and mode-selective vibrational autodetachment, in comparison with traditional non-resonant PES. Combining photodetachment spectroscopy and resonant PES, we are able to determine eleven fundamental vibrational frequencies of the N1[T–H]˙ radical in the low frequency regime, including the four lowest-frequency out-of-plane modes.

## 2. Experimental method

Our high-resolution PE imaging apparatus^53^ is equipped with an electrospray ionization (ESI) source,^54^ a temperature-controlled cryogenic ion trap^55,56^ and a time-of-flight mass spectrometer. This is an improved version of our original ESI-PES apparatus.^57,58^ One of the advantages of PE imaging is that photoelectron angular distributions (PADs) are obtained at the same time,^59,60^ providing information about the nature of the occupied molecular orbital from which the electron is ejected. The deprotonated thymine anions were generated by electrospray of a 1 mM solution of thymine (from Sigma Aldrich) dissolved in a mixed water–methanol solvent (1 : 9 volume ratio) at a pH of ∼8. The anions from the ESI source were directed by two radio-frequency (RF) quadrupole ion guides and one RF-octopole ion guide into a cryogenically-cooled three-dimensional Paul trap in a more compact linear configuration^61^ than our first generation cold ion trap.^55^ After being accumulated and cooled *via* collisions with a He/H_2_ buffer gas (4 : 1 volume ratio) in the ion trap for 0.1 s, the anions were pulsed out into the extraction zone of a time-of-flight mass spectrometer. The desired anions were selected by a mass gate and focused into a co-linear velocity map imaging apparatus,^53^ where anions were photodetached by a linearly polarized laser beam. The laser polarization was aligned parallel to the imaging detector. Photoelectrons were projected onto a position-sensitive detector consisting of a pair of 75 mm diameter micro-channel plates coupled to a phosphor screen and captured by a charge-coupled-device camera. The recorded two-dimensional PE images were symmetrized and inverse-Abel transformed to obtain three-dimensional photoelectron distributions. This reconstruction was carried out by both the pBASEX^62^ and BASEX programs,^63^ which gave similar results.

The velocity map imaging apparatus was calibrated with the PE images of atomic Au^–^ at several photon energies.^57^ Two photodetachment laser systems were used: a Nd : YAG laser and a Nd : YAG pumped tunable dye laser (Δ*λ* ∼ 0.0015 nm, Sirah Cobra-Stretch). The lowest temperature that our cold ion trap can achieve is 4.4 K, measured by a thermal couple off the outer wall of the Paul trap.^61^ The experiments reported in the current work were all performed by operating the ion trap at 4.4 K to achieve the best cooling. The spectral resolution was 3.8 cm^–1^ for 55 cm^–1^ kinetic energy (KE) electrons and about 1.5% (ΔKE/KE) for KE above 1 eV.

## 3. Results

### 3.1. The non-resonant photoelectron image and spectrum of [T–H]^–^ at 354.84 nm

**Fig. 2:**
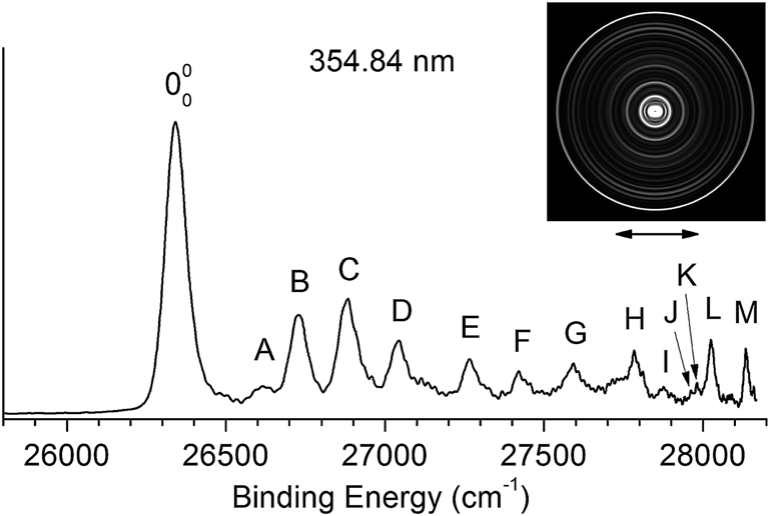
The non-resonant photoelectron image and spectrum of N1[T–H]^–^ at 354.84 nm. The double arrow below the image indicates the direction of the laser polarization.

Fig. 2 shows the traditional non-resonant PE image and spectrum of [T–H]^–^ at 354.84 nm, representing the detachment transition from the [T–H]^–^ anion to the electronic ground state of the [T–H]˙ radical. Since vibrational hot bands are completely eliminated, the intense lowest binding energy peak should correspond to the 000 transition. Numerous peaks, labeled as A–M, are resolved at higher binding energies, which should correspond to the excited vibrational levels of the [T–H]˙ radical. The current PE spectrum at 354.84 nm is considerably better-resolved than in the previous study,^23^ as compared in Fig. S1.† As will be discussed later and shown in Fig. S1,† the observed spectrum is unambiguously due to the N1[T–H]^–^ isomer. The PE image in Fig. 2 shows that the PAD has s + d character, implying that the highest occupied molecular orbital of N1[T–H]^–^ is a p-type orbital. The binding energies, shifts from the 000 peak, and assignments of the observed peaks are summarized in Table 1. The binding energies of some of the peaks, including the 000 transition, are from the higher resolution resonant PE spectra to be presented later. The assignments are done using both the photodetachment spectrum and the resonant PE spectra, as well as by comparing with theoretical calculations (Tables 2 and S1†).

**Table 1:** The binding energies (BEs), shifts relative to the 000 peak and assignments of vibrational peaks observed for the deprotonated thymine radical (N1[T–H]˙) from both traditional and resonant-enhanced photoelectron spectroscopy of deprotonated thymine anions (N1[T–H]^–^). Peaks 000 and A–M correspond to transitions observed in the non-resonant spectrum shown in Fig. 2. Peaks a–h were measured from the resonant-enhanced spectra in Fig. 4 and 5

Peak	BE^*a*^ (cm^–1^)	Shift (cm^–1^)	Assignment
000	26 322(5)	0	
A	26 604(5)	282	5_0_^1^
B	26 715(5)	393	7_0_^1^
C	26 884(25)	562	9_0_^1^
D	27 044(22)	722	12_0_^1^
E	27 267(18)	945	7_0_^1^9_0_^1^
F	27 422(16)	1100	9_0_^2^
G	27 593(14)	1271	9_0_^1^12_0_^1^
H	27 784(12)	1462	7_0_^1^9_0_^2^
I	27 873(12)	1551	5_0_^1^9_0_^1^12_0_^1^
J	27 963(10)	1641	9_0_^3^
K	27 983(10)	1661	7_0_^1^9_0_^1^12_0_^1^
L	28 024(8)	1702	5_0_^1^12_0_^2^
M	28 135(5)	1813	9_0_^2^12_0_^1^
α	26 282(8)	–40	?^*b*^
a	26 396(8)	74	1_0_^1^
b	26 413(5)	91	2_0_^1^
c	26 462(5)	140	3_0_^1^
d	26 482(5)	160	1_0_^1^2_0_^1^
e	26 526(5)	204	1_0_^1^3_0_^1^
f	26 582(5)	260	4_0_^1^
g	26 644(8)	321	1_0_^1^4_0_^1^
h	26 678(5)	356	2_0_^1^4_0_^1^

^*a*^Numbers in parentheses indicate the experimental uncertainties in the last digit. The binding energies for peaks C–M were measured from the non-resonant spectrum in Fig. 2 and all other peaks were from the higher resolution resonant spectra in Fig. 4 and 5.

^*b*^No satisfactory explanation is found for this feature observed in Fig. 5b.

**Table 2:** Comparison of the calculated vibrational frequencies for the thirteen lowest-frequency modes of N1[T–H]˙ with experimental values from the current work. The unscaled harmonic frequencies and the infrared intensities were calculated using the B3LYP/6-311++G(d,p) method

Mode^*a*^	Symmetry	Theo. (cm^–1^)	Exp.^*b*^ (cm^–1^)	Peak^*c*^	IR intensity (km mol^–1^)
ν_1_	A′′	70	74(8)		0.43
ν_2_	A′′	93	**92(5)**	w	0.43
ν_3_	A′′	135	140(5)		6.64
ν_4_	A′′	262	**260(5)**	1	1.58
ν_5_	A′	285	**283(5)**	2	2.40
ν_6_	A′′	388	**398(5)**	6	3.92
ν_7_	A′	397	**390(5)**	5	18.98
ν_8_	A′	450			12.55
ν_9_	A′	555	**547(5)**	10	1.16
ν_10_	A′	608	**602(5)**	11	2.17
ν_11_	A′′	670			63.91
ν_12_	A′	720	**714(5)**	15	3.22
ν_13_	A′′	727	**718(5)**	16	9.81

^*a*^The normal modes are enumerated according to the calculated frequencies in increasing order.

^*b*^Numbers in parentheses indicate the experimental uncertainties in the last digit. The experimental values in bold face were measured from the photodetachment spectrum in Fig. 3 and those in normal font for ν_1_ and ν_3_ were obtained from the resonant photoelectron spectra in Fig. 4 and 5.

^*c*^The labels are from Fig. 3 and indicate the peaks from which the corresponding vibrational frequencies were measured. Also see Table 3.

### 3.2. Photodetachment spectroscopy and observation of dipole-bound states in N1[T–H]^–^

**Fig. 3:**
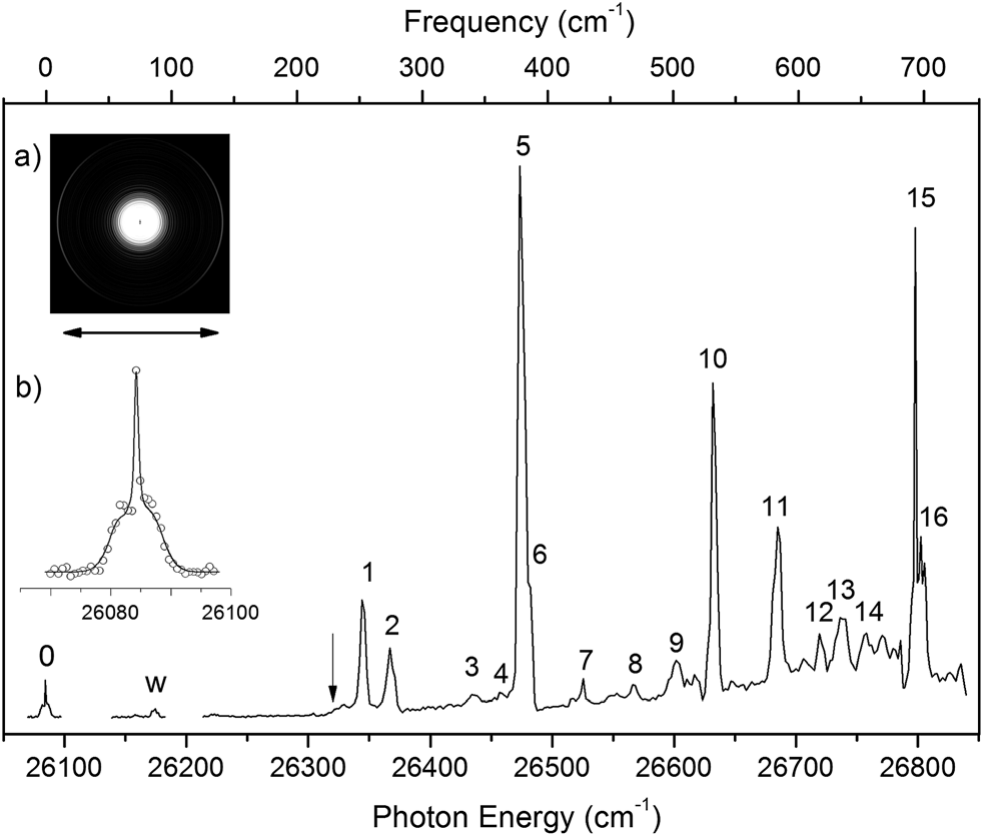
The photodetachment spectrum of N1[T–H]^–^ obtained by measuring the total electron yield as a function of laser wavelength across the detachment threshold. The arrow at 26 322 cm^–1^ indicates the detachment threshold. The peaks labeled as 1–16 are due to autodetachment from vibrational levels of the DBS of N1[T–H]^–^ while the peaks labeled as 0 and w are due to single-color resonant two-photon detachment. Peak 0 at 26 084 cm^–1^ represents the dipole-bound ground state, corresponding to the outer ring of the photoelectron image in inset (a). The double arrow below the image indicates the direction of the laser polarization. Inset (b) shows a rotational simulation for the 0 peak (c-type) with a rotational temperature of 35 K (dot: experimental data; line: simulation).

**Fig. 4:**
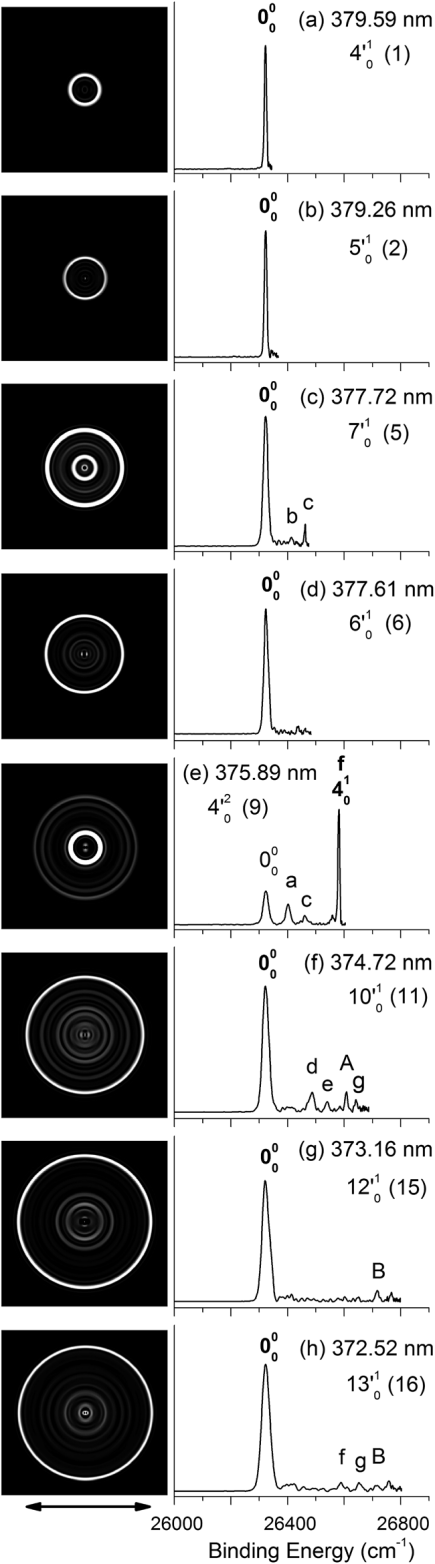
Resonant photoelectron images and spectra of N1[T–H]^–^ at eight detachment wavelengths, corresponding to the resonance peaks in Fig. 3. The peak number (in parentheses) and the single-mode vibrational levels of the DBS excited state are given in each spectrum. The double arrow below the images indicates the direction of the laser polarization. The labels in capital letters are the same as in Fig. 2 and those in bold face indicate the autodetachment-enhanced final vibrational states.

By measuring the total electron yield as a function of photon energy across the detachment threshold, we obtained a photodetachment spectrum for N1[T–H]^–^, as shown in Fig. 3, which consisted of scans using several dyes. The spectrum above 26 200 cm^–1^ was scanned with a step size of 0.03 nm, whereas the two scans below 26 200 cm^–1^ were done with a finer step of 0.01 nm. The arrow at 26 322 cm^–1^ indicates the detachment threshold, which was determined more accurately from the slow-electron resonant PE spectra in Fig. 4a and b. The overall baseline above the threshold represents the intensities of direct non-resonant detachment signals from the ground state of N1[T–H]^–^ to the neutral final state. The non-resonant signals are continuous and slowly increase with photon energy as more detachment channels open up. In addition, sixteen sharp peaks above the detachment threshold are observed and labeled as 1–16. These sharp peaks provide evidence for the existence of a DBS;^35–37^ they are due to autodetachment from vibrational levels of the DBS of N1[T–H]^–^.

**Fig. 5:**
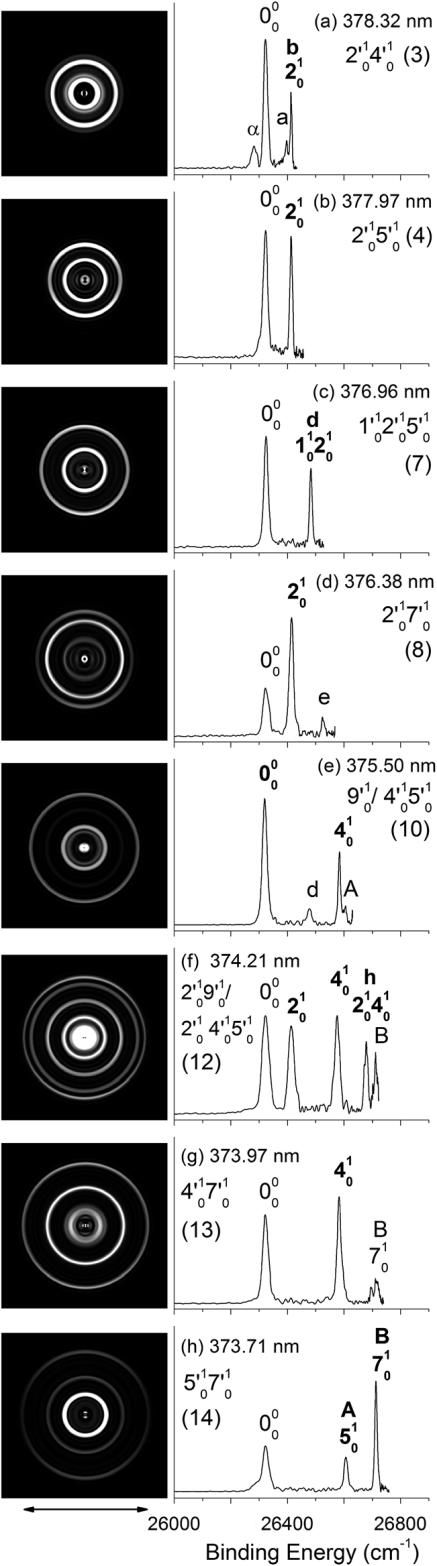
Resonant photoelectron images and spectra of N1[T–H]^–^ at eight detachment wavelengths, corresponding to the resonance peaks in Fig. 3. The peak number (in parentheses) and the combinational vibrational levels of the DBS excited state are given in each spectrum. The double arrow below the images indicates the direction of the laser polarization. The labels in capital letters are the same as in Fig. 2 and those in bold face indicate the autodetachment-enhanced final vibrational states.

Below the detachment threshold, we observed two weak peaks (labeled as 0 and w), which are due to single-color two-photon detachment. The peak 0 at 26 084 cm^–1^ was determined to be the vibrational ground state of the DBS, corresponding to the outer ring of the PE image shown in inset (a). The PAD of the PE image exhibits p-wave character, indicating that the dipole-bound electron can be viewed as in an s-type orbital, as was also observed in ground-state dipole-bound anions previously.^52^ The binding energy of the DBS refers to the energy difference between the neutral ground state and the vibrational ground state of the DBS, and it is measured accurately to be 238 ± 5 cm^–1^. This binding energy is quite high, relative to the 146 cm^–1^ binding energy of the DBS observed for the deprotonated uracil anion,^50^ suggesting that the N1[T–H]˙ radical has a larger dipole moment than N1[U–H]˙. Peak w corresponds to a vibrational level of the DBS, which is below the detachment threshold and can only be accessed *via* a two-photon process. The energies of the observed vibrational levels of the DBS can be obtained readily with respect to the DBS ground state, as shown in the top scale of Fig. 3. Table 3 summarizes the photon energies, shifts from the DBS ground state, and assignment of the observed vibrational peaks. The assignments for peaks 1–16 are all supported by and based on resonant PE spectra to be presented next in Fig. 4 and 5 and the calculated frequencies in Table 2.

**Table 3:** The excitation photon energies (PEs), shifts from peak 0 (the ground vibrational level of the dipole-bound state of N1[T–H]^–^) and assignments of the observed vibrational autodetachment resonances in Fig. 3. The PEs were measured from the peak maxima

Peak	PE^*a*^ (cm^–1^)	Shift (cm^–1^)	Assignment
0	26 084(5)	0	DB ground state
w	26 176(5)	92	2′_0_^1^
1	26 344(5)	260	4′_0_^1^
2	26 367(5)	283	5′_0_^1^
3	26 432(5)	348	2′_0_^1^4′_0_^1^
4	26 457(5)	373	2′_0_^1^5′_0_^1^
5	26 474(5)	390	7′_0_^1^
6	26 482(5)	398	6′_0_^1^
7	26 528(5)	444	1′_0_^1^2′_0_^1^5′_0_^1^
8	26 569(5)	485	2′_0_^1^7′_0_^1^
9	26 603(5)	519	4′_0_^2^
10	26 631(5)	547	9′_0_^1^/4′_0_^1^5′_0_^1^
11	26 686(5)	602	10′_0_^1^
12	26 723(5)	639	2′_0_^1^9′_0_^1^/2′_0_^1^4′_0_^1^5′_0_^1^
13	26 740(5)	656	4′_0_^1^7′_0_^1^
14	26 759(5)	675	5′_0_^1^7′_0_^1^
15	26 798(5)	714	12′_0_^1^
16	26 802(5)	718	13′_0_^1^

^*a*^Numbers in parentheses indicate the experimental uncertainties in the last digit.

All the vibrational peaks in Fig. 3 are rotationally broadened. This is seen more clearly from the higher-resolution ground state peak 0. We performed a rotational simulation using the PGOPHER program,^64^ as shown in inset (b) of Fig. 3, and obtained a rotational temperature of 35 K. This rotational temperature is similar to that obtained in our previous study on N1[U–H]^–^ when the ion trap was operated at 4.4 K.^50^ A recent study on the simpler acetate anion shows a rotational temperature of ∼20 K at an ion trap temperature of 4.4 K.^65^

### 3.3. Resonant photoelectron images and spectra of N1[T–H]^–^

By tuning the detachment laser to the above-threshold vibrational peaks (1–16) observed in Fig. 3, we obtained sixteen high-resolution resonantly-enhanced PE images and spectra, as shown in Fig. 4 and 5, where the DBS vibrational level (ν′_*x*_) and the corresponding peak number labeled in Fig. 3 are given in each spectrum. Two detachment processes contributed to these spectra: the direct non-resonant photodetachment signals and the resonantly-enhanced vibrational autodetachment *via* the DBS. The resonant enhancement can be appreciated from the peak intensities relative to the baseline at each photon energy in Fig. 3. The resonant PE spectra are highly non-Franck–Condon: some vibrational peaks are dramatically enhanced, indicating that they are the final vibrational states of the neutral radical from vibrational state-selective autodetachment *via* the DBS. The eight spectra shown in Fig. 4 each contain autodetachment from vibrational levels of the DBS involving a single vibrational mode, whereas those given in Fig. 5 involve autodetachment from combination or overlapping vibrational levels of the DBS. The assignments in bold face in each spectrum in Fig. 4 and 5 indicate the major enhanced vibrational peaks. Moreover, numerous Franck–Condon inactive or unresolved peaks in Fig. 2 were observed in the resonant spectra, labeled using lower case letters (a–h). The binding energies, shifts from the 000 peak, and assignments of peaks a–h are also given in Table 1.

## 4. Discussion

### 4.1. The observed [T–H]^–^ isomer and assignment of the non-resonant PE spectrum

The EA of 3.250 ± 0.015 eV obtained by Parsons *et al.*^23^ was consistent with the computed EA (3.2–3.4 eV) for the N1[T–H]˙ isomer.^18–22^ To further ascertain their observed isomer, they did Franck–Condon simulations for both the N1[T–H]^–^ and N3[T–H]^–^ isomers and found that the N1[T–H]^–^ isomer was in agreement with the observed spectrum. In Fig. S1,† we compare the current high resolution spectrum, also at 354.84 nm, with the previous spectrum and the Franck–Condon simulation for the N1[T–H]^–^ isomer by Parsons *et al.* Clearly, the previous Franck–Condon simulation agrees very well with the current spectrum. The simulated Franck–Condon profile and the vibrational frequencies for the N3[T–H]˙ isomer were quite different.^23^ Hence, there is no question that the observed isomer in both the current and the previous experiments was the N1[T–H]^–^ isomer. The calculated EA for the N3[T–H]˙ isomer (3.7–4.5 eV)^19,21^ was higher than the photon energy used (354.84 nm). Hence, even if the N3[T–H]^–^ isomer were present in our anion beam, it would not be accessible. Therefore, all the vibrational features resolved in Fig. 2 should be due to the ground electronic state of the N1[T–H]˙ radical final state.

To help with the assignment of the observed vibrational peaks, we carried out density functional theory calculations at the B3LYP/6-311++(d,p) level for the vibrational frequencies of N1[T–H]˙. There are thirty-six normal modes for N1[T–H]˙ and the complete list of the calculated fundamental vibrational frequencies is given in Table S1† in increasing order of frequencies in the ESI.† The thirteen lowest frequency modes are shown in Fig. S2† and their calculated frequencies are reproduced in Table 2 to be compared with the current experimental measurements. In the non-resonant PE spectrum, only symmetry-allowed modes with significant Franck–Condon factors can be observed. Because both N1[T–H]^–^ and N1[T–H]˙ have *C*_s_ symmetry, only in-plane vibrational modes (A′) or even quanta of out-of-plane modes (A′′) are symmetry-allowed. As shown in Table 2 and Fig. S2,† modes such as ν_5_, ν_7_–ν_10_, and ν_12_ are all in-plane modes. By comparing the theoretical frequencies and the experimental shifts from the 000 peak in Table 1, we can readily assign the four vibrational peaks (A, B, C and D) in Fig. 2 to 5_0_^1^, 7_0_^1^, 9_0_^1^, and 12_0_^1^, respectively. Peaks F and J can be assigned to the second and third overtones of the ν_9_ mode. The remaining peaks resolved in Fig. 2 can all be assigned to combinational vibrational levels of modes ν_5_, ν_7_, ν_9_, and ν_12_, as summarized in Table 1. It is seen that peaks 000, C (9_0_^1^), F (9_0_^2^), and J (9_0_^3^) consist of the main vibrational progression of the ν_9_ mode, which represents the most active mode upon electron detachment from N1[T–H]^–^. Modes ν_7_ and ν_12_ are also quite active, judging by the significant intensities of the 7_0_^1^ (B) and 12_0_^1^ (D) peaks, and there may be unresolved overtones of these modes, as well.

It should be pointed out that the vibrational frequencies for all the observed modes are measured more accurately from the photodetachment spectrum and the resonant PE spectra to be presented next.

### 4.2. The nature and assignment of the photodetachment spectrum

The photodetachment spectrum in Fig. 3 covers a spectral range of ∼700 cm^–1^ above the DBS ground state. All the photodetachment signals should come from the N1[T–H]^–^ isomer. The resonant peaks in Fig. 3 represent the vibrational levels of the DBS of N1[T–H]^–^. The above-threshold peaks (1–16) in Fig. 3 should display Fano-type asymmetric lineshapes,^66^ due to the interference between the direct non-resonant detachment and autodetachment *via* the DBS. This asymmetric lineshape is seen more clearly on the higher energy side of peak 5/6 near 26 500 cm^–1^ in Fig. 3, whereas the below-threshold peaks (0 and w) do not display such asymmetry. The intensities of the above-threshold peaks (1–16) relative to the continuous baseline at each photon energy represent the resonant enhancement *via* autodetachment from the given vibrational level of the DBS. As we showed previously,^47,50,65^ the vibrational frequencies of the DBS of the anion were the same as the corresponding neutral radical within our experimental uncertainty. For example, comparing Tables 1 and 3, we can readily see that the vibrational peaks A, B, C, and D resolved in the PE spectrum (Fig. 2) correspond to the same vibrational modes of the DBS represented by peaks 2, 5, 10, and 15 (Fig. 3), respectively. Therefore, the vibrational frequencies of N1[T–H]˙ can be obtained more accurately from the photodetachment spectrum in Fig. 3. Indeed, most of the vibrational frequencies obtained for the N1[T–H]˙ radical in the current study are from the photodetachment spectrum, as given in Table 2, where only the frequencies of the ν_1_ and ν_3_ modes were measured from the resonant PE spectra in Fig. 4 and 5.

More importantly, many more vibrational peaks were resolved in the photodetachment spectrum because of the high spectral resolution and vibrational cooling, in comparison to the PE spectrum in Fig. 2. Among these peaks, 1 (4′_0_^1^), 6 (6′_0_^1^), 11 (10′_0_^1^), and 16 (13′_0_^1^) are relatively strong and they correspond to four new vibrational modes (Table 3), including the symmetry-forbidden modes, ν_4_(A′′), ν_6_(A′′), and ν_13_(A′′). The relatively high intensities of these peaks can be due to either strong vibronic couplings or even a slight out-of-plane distortion of the neutral core. Peak 6 is only 8 cm^–1^ higher than the strong peak 5 and appeared as a shoulder on the higher energy side of peak 5 (Fig. 3). The frequency represented by peak 6 (398 cm^–1^) is in better agreement with the calculated frequency of the ν_7_ mode, as shown in Table 2. However, peak 6 is much weaker than peak 5 and is consistent with a symmetry forbidden transition, whereas the strong peak 5 is consistent with the ν_7_ mode, which has a significant Franck–Condon factor as revealed in the non-resonant PE spectrum (peak B in Fig. 2). The remaining eight peaks are quite weak and they all consist of combinational vibrational levels of the DBS (Table 3). Most of these weak peaks are symmetry-forbidden and they were observed partly due to the resonant enhancement and partly due to strong vibronic coupling effects, as also observed in high-resolution PES studies.^67,68^ As will be shown below, many of the assignments for these combination vibrational levels are confirmed by the resonant PE spectra to be discussed below, *via* autodetachment enhancement.

The weak peak w is only 92 cm^–1^ above the DBS ground state, corresponding to a very low frequency mode. The measured frequency is in excellent agreement with the calculated value for the ν_2_(A′′) mode (Table 2). This frequency is below the detachment threshold and peak w was due to a two-photon detachment process, similar to the DBS ground state. The DBS ground state can be determined from the resonant PE spectra if the excited vibrational modes of the DBS are known. The direct observation of the DBS ground state *via* the two-photon process in Fig. 3 at 26 084 ± 5 cm^–1^ provides the most accurate measurement for the binding energy of the DBS as 238 ± 5 cm^–1^ relative to the detachment threshold. This DBS binding energy is quite high, in comparison to that observed for the deprotonated uracil anion (146 cm^–1^),^47^ suggesting that the N1[T–H]˙ radical has a higher dipole moment than the N1[U–H]˙ radical.

### 4.3. Resonant PE spectra *via* vibrational state-selective autodetachment of the DBS

Because the extra electron in the DBS has very little effect on the molecular core, the anion structure in the DBS is nearly identical to that of the neutral structure, analogous to Rydberg states. Therefore, under the harmonic approximation, each vibrational level of the DBS is autodetached to the nearest neutral vibrational level, during which one vibrational quantum in the DBS is coupled to the electron to cause detachment. This gave rise to the so-called Δ*v* = –1 vibrational propensity rule,^48^ which was first developed for autoionization from Rydberg states.^49^ Basically, an excitation to the ν′_*x*_^*n*^ vibrational level of the DBS can only autodetach to the ν_*x*_^*n*–1^ vibrational level of the corresponding mode in the neutral final state, resulting in a highly non-Franck–Condon PE spectrum. The Δ*v* = –1 vibrational propensity rule was observed in our previous high-resolution resonant PES experiment on the phenoxide anion and photodetachment of the deprotonated uracil anion.^47,50^ It is also observed in a recent study of the acetate anion.^65^

**Fig. 6:**
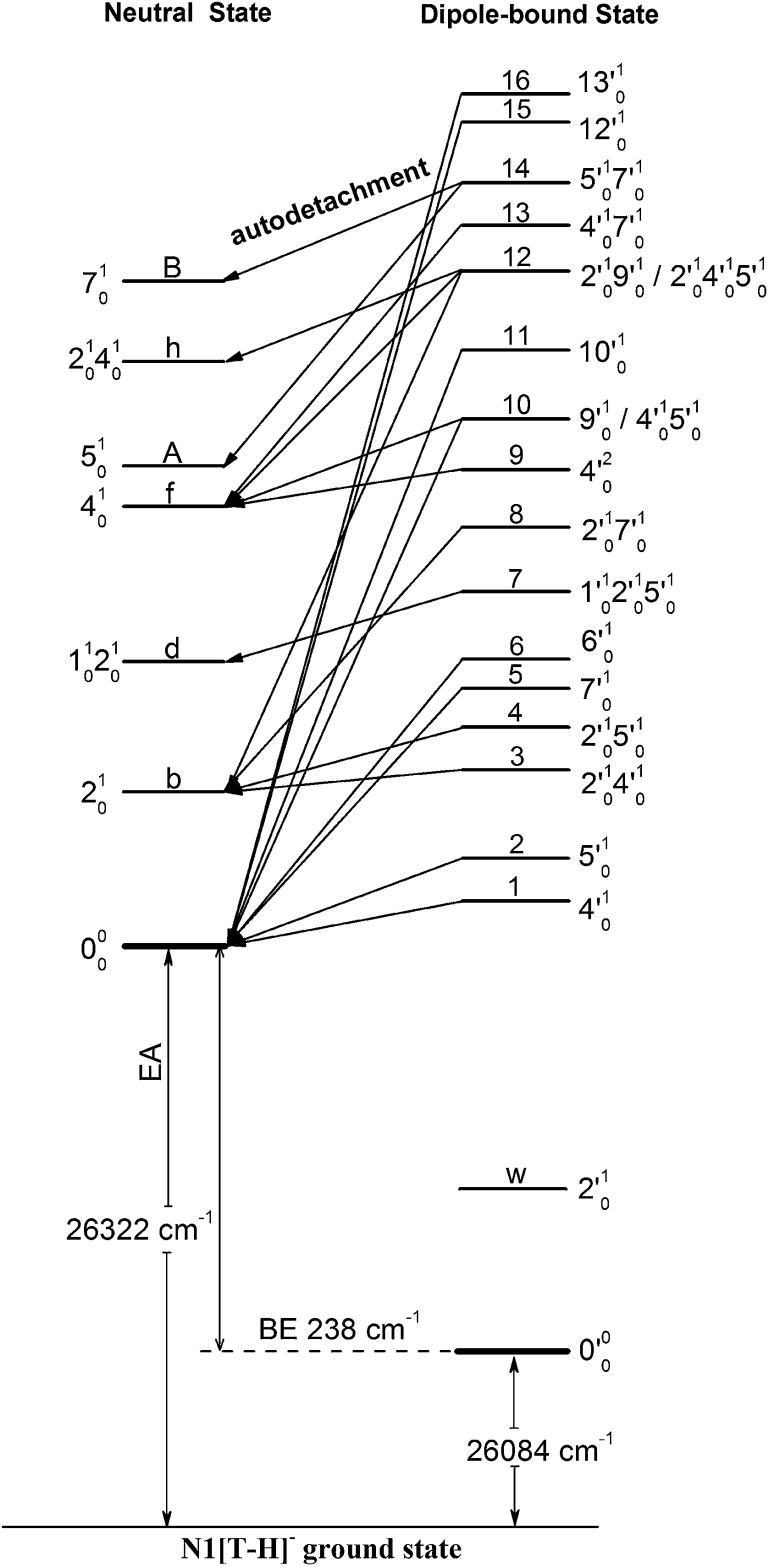
Schematic energy level diagram for direct detachment to the vibrational levels of the N1[T–H]˙ radical (left) and autodetachment from the vibrational levels of the DBS of N1[T–H]^–^ (right). The EA of N1[T–H]˙ and the binding energy of the DBS are indicated. Autodetachment from the vibrational levels of the DBS to the neutral final states is indicated by the arrows. The vibrational levels of the DBS labeled with 1–16 correspond to peaks 1–16 in Fig. 3 and the neutral states labeled with letters are the same as those in Fig. 2, 4, 5, and Table 1.

Fig. 4a–d and f–h all show that the 000 peak is significantly enhanced, suggesting autodetachment from a fundamental excitation of a single vibrational mode (ν′_*x*_^1^) of the DBS of N1[T–H]^–^. The PADs of the 000 peak in the corresponding PE images are all isotropic, different from the s + d wave-like PAD shown in the non-resonant spectrum in Fig. 2. This observation is consistent with an indirect detachment process, implying that the lifetime of the autodetaching state is longer than the molecular rotational period. As indicated in each spectrum and in Table 3, the photon energies used for Fig. 4a–d and f–h corresponded to excitations to the 4′_0_^1^, 5′_0_^1^, 7′_0_^1^, 6′_0_^1^, 10′_0_^1^, 12′_0_^1^ and 13′_0_^1^ levels of the DBS, respectively. The autodetachment processes from these DBS vibrational levels are shown schematically in Fig. 6. Fig. 4e shows that the 4_0_^1^ vibrational peak is significantly enhanced, indicating that the autodetachment is from the 4′_0_^2^ overtone, as shown in Table 3 and Fig. 6, obeying the Δ*v* = –1 vibrational propensity rule.

Apart from the enhanced peaks discussed above, several weak peaks were also observed in Fig. 4, which were not present or resolved in Fig. 2. These peaks are labeled with lower case letters in Fig. 4; their binding energies and assignments are also given in Table 1. The assignments of these weak peaks were done by comparing the observed vibrational energies with the calculated vibrational frequencies. Many of these vibrational excitations are not allowed by symmetry and probably borrowed intensity due to vibronic couplings. The peaks a, b, and c are of particular interest, because they give us the fundamental frequencies of the three lowest frequency modes (ν_1_, ν_2_, and ν_3_) for N1[T–H]˙ (Tables 1 and 2). The ν_2_ mode was also observed in the photodetachment spectrum, represented by peak w (Fig. 3 and Table 2), as discussed above.

### 4.4. Resonant PE spectra *via* state- and mode-selective autodetachment of the DBS

In cases of resonant excitations to combinational vibrational levels of the DBS (ν′_*x*_^*n*^ν′_*y*_^*m*^), the final neutral level can be either ν_*x*_^(*n*–1)^ν_*y*_^*m*^ or ν_*x*_^*n*^ν_*y*_^(*m*–1)^. Hence, not only is there a vibrational state-selectivity due to the Δ*v* = –1 propensity rule, there is also a mode-specificity in the autodetachment process, resulting in highly non-Franck–Condon and interesting resonant PE spectra, as shown in Fig. 5.

Fig. 5a–d all show only one enhanced vibrational peak due to autodetachment from a combinational vibrational level of the DBS of N1[T–H]^–^, *i.e.*, the 2′_0_^1^4′_0_^1^, 2′_0_^1^5′_0_^1^, 1′_0_^1^2′_0_^1^5′_0_^1^ and 2′_0_^1^7′_0_^1^ vibrational levels, respectively. These DBS levels autodetached to the nearest neural vibrational levels, 2_0_^1^, 2_0_^1^, 1_0_^1^2_0_^1^ and 2_0_^1^, respectively, as shown schematically in Fig. 6. The PADs of the corresponding enhanced peaks in their PE images are, again, all isotropic. The different PADs for direct photodetachment and autodetachment can be seen most vividly in Fig. 5b–d, where the 000 peak exhibits s + d wave character because it comes from direct non-resonant photodetachment. An interesting common feature of these four vibrational levels of the DBS is that in each case only one vibrational mode has high enough vibrational energy for autodetachment because of the relatively large binding energy of the DBS (238 cm^–1^). Hence, only one autodetachment channel is available in each of the four cases. The binding energy for the 2_0_^1^ final vibrational state of the neutral species can be accurately measured in Fig. 5a and b. The vibrational frequency of the ν_2_ mode can be measured equally accurately from these data as the w peak in the photodetachment spectrum (Tables 2 and 3).

The weak a and e peaks were also observed in Fig. 5a and d, respectively. Surprisingly, a weak peak, labeled as α, was observed in Fig. 5a, which has a binding energy 40 cm^–1^ smaller than that of the 000 peak (Table 1). The only possible explanation for this peak would be due to a vibrational hot band of the N1[T–H]^–^ anion. However, the 40 cm^–1^ separation between peak α and the 000 peak is too small to correspond to any vibrational level of the anion. Furthermore, no other spectrum showed any sign of vibrational hot bands because of the excellent vibrational cooling in our cold ion trap. Thus, we do not have a good explanation for this peak, although we found that it was reproducible.

Fig. 5e displays the resonant PE spectrum when the detachment laser was tuned to peak 10 in the detachment spectrum corresponding to the 9′_0_^1^ level of the DBS (Table 3). The ν_9_ mode is the most active mode observed in the non-resonant PE spectrum in Fig. 2. We expected to observe an enhanced 000 transition from autodetachment from the 9′_0_^1^ DBS level. However, we also observed that the 4_0_^1^ final state was significantly enhanced, which must come from autodetachment of a combinational vibrational level involving the ν′_4_ mode. The 4′_0_^1^5′_0_^1^ level has an excitation energy (543 cm^–1^), very close to that of 9′_0_^1^ (547 cm^–1^), and peak 10 at 375.50 nm must contain excitation to both vibrational levels. The coupling of the ν′_5_ quantum to the DBS electron produced the enhanced 410 final state in Fig. 5e. The ν′_4_ quantum (260 cm^–1^) is also larger than the binding energy of the DBS, but we did not see autodetachment induced by the ν′_4_ mode. Such mode selectivity was also observed previously in the phenoxide case,^47^ suggesting that the ν′_5_ mode has stronger coupling to the DBS electron than the ν′_4_ mode.

Fig. 5f shows a very complicated resonant PE spectrum with three autodetachment-enhanced peaks corresponding to the 2_0_^1^, 4_0_^1^, and 2_0_^1^4_0_^1^ final neutral states, suggesting again excitations to overlapping vibrational levels of the DBS at 374.21 nm (peak 12). The enhanced 2_0_^1^ final state must come from autodetachment of the 2′_0_^1^9′_0_^1^ combinational DBS level, whereas the observation of the 2_0_^1^4_0_^1^ final state suggests autodetachment from the 2′_0_^1^4′_0_^1^5′_0_^1^ combinational level, based on the vibrational frequencies presented in Table 2. Again, we see stronger coupling of the ν′_5_ mode to produce the 2_0_^1^4_0_^1^ final neutral state, whereas the coupling of the ν′_4_ mode is weak, even though it cannot be ruled out because the 2_0_^1^5_0_^1^ final state may contribute to the near-threshold peak B in Fig. 5f. The enhancement of the 4_0_^1^ final state is surprising because it could only come from autodetachment from the 2′_0_^1^4′_0_^1^5′_0_^1^ level by coupling two vibrational quanta (2′_0_^1^5′_0_^1^) to the outgoing electron, violating the Δ*v* = –1 propensity rule, which could be an indication of anharmonic effects.^48,49^

In Fig. 5g, the 4_0_^1^ final state is enhanced, indicating that the 4′_0_^1^7′_0_^1^ DBS level was excited at 373.97 nm. The fact that the 7_0_^1^ final state (peak B) was not enhanced at all suggested that the ν′_7_ mode has much stronger coupling with the outgoing electron than the ν′_4_mode. In Fig. 5h, we observe that the 5_0_^1^ and 7_0_^1^ final states are enhanced in comparison with Fig. 2, suggesting that the 5′_0_^1^7′_0_^1^ DBS level is excited at 373.71 nm. In this case, the intensity of the 5_0_^1^ final state seems to be enhanced slightly more than the 7_0_^1^ final state, suggesting that the ν′_7_ mode has stronger coupling with the outgoing electron.

### 4.5. Low frequency vibrational modes of N1[T–H]˙ and mode-dependent vibronic coupling

As shown in Table 2, we obtained eleven of the lowest thirteen vibrational frequencies for the deprotonated thymine radical, N1[T–H]˙. The thirteen normal modes are shown in Fig. S2.† The two lowest-frequency modes (ν_1_ and ν_2_) involve the internal rotations of the CH_3_ group, as shown in Fig. S2.† Such low frequency vibrations are quite difficult to measure using infrared spectroscopy techniques. The most Frank–Condon-active mode ν_9_ is a ring deformation mode, which was also revealed from the previous Franck–Condon simulation (Fig. S1†).^23^

Simons developed theoretical frameworks for computing vibrational autodetachment rates.^48^ Lineberger and co-workers observed vibrational dependence of autodetachment rates in H_2_CCC^–^.^46^ The mode preference of autodetachment has been observed from the combinational vibrational levels of phenoxide.^47^ The autodetachment process involves coupling of vibrational motions with electronic degrees of freedom. The vibronic coupling is related to the vibration-induced dipole moment change, which is similar to the IR intensity. Thus, we can use IR intensities to qualitatively understand the mode preference in the autodetachment involving combinational vibrational levels. In Table 2, we give the computed IR intensities for the thirteen vibrational modes. Among the eleven observed vibrational modes, we note that the ν_7_ mode, which exhibits the strongest vibronic coupling, has the strongest IR intensity, in agreement with the observed mode preference in Fig. 5g and h. The IR intensity of the ν_5_ mode is stronger than that of the ν_4_ mode, consistent with the mode preference observed in Fig. 5e and f. The current work provides detailed vibrational and mode-dependent information for autodetachment from the DBS of N1[T–H]^–^, which would be an interesting system with which to further investigate vibronic couplings.

## 5. Conclusions

In conclusion, we report a photodetachment and high-resolution photoelectron imaging study of cold thymine deprotonated at its N1 position, N1[T–H]^–^. The electron affinity of the N1[T–H]˙ radical was accurately measured to be 26 322 ± 5 cm^–1^ (3.2635 ± 0.0006 eV). A dipole-bound state of N1[T–H]^–^ was observed with a binding energy of 238 ± 5 cm^–1^ below the detachment threshold. Photodetachment spectroscopy revealed eighteen vibrational levels of the dipole-bound states of the N1[T–H]^–^ anion. Sixteen of the vibrational levels were above the detachment threshold and led to sixteen high-resolution autodetachment-enhanced resonant photoelectron spectra, which contained much richer vibrational information than traditional photoelectron spectroscopy. Resonant excitations to the dipole-bound states led to the observation of low-frequency symmetry-forbidden vibrational modes, which would otherwise be difficult to access. In total, eleven fundamental vibrational frequencies were obtained for the N1[T–H]˙ radical in the low frequency regime, including the four lowest-frequency out-of-plane modes.

## Supplementary Material

Supplementary information

## References

[cit1] Boudaiffa B., Cloutier P., Hunting D., Huels M. A., Sanche L. (2000). Science.

[cit2] Faraji S., Groenhof G., Dreuw A. (2013). J. Phys. Chem. B.

[cit3] Faraji S., Dreuw A. (2014). Annu. Rev. Phys. Chem..

[cit4] Simons J. (2006). Acc. Chem. Res..

[cit5] Kim S., Wheeler S. E., Schaefer H. F. (2006). J. Chem. Phys..

[cit6] Thicoipe S., Carbonniere P., Pouchan C. (2013). Phys. Chem. Chem. Phys..

[cit7] Michaud M., Bazin M., Sanche L. (2012). Int. J. Radiat. Biol..

[cit8] Denifl S., Sulzer P., Zappa F., Moser S., Krautler B., Echt O., Bohme D. K., Mark T. D., Scheier P. (2008). Int. J. Mass Spectrom..

[cit9] Baccarelli I., Bald I., Gianturco F. A., Illenberger E., Kopyra J. (2011). Phys. Rep..

[cit10] Alizadeh E., Sanz A. G., Madugundu G. S., Garcia G., Wagnera J. R., Sanche L. (2014). Radiat. Res..

[cit11] King S. B., Yandell M. A., Neumark D. M. (2013). Faraday Discuss..

[cit12] Ptasinska S., Denifl S., Scheier P., Illenberger E., Mark T. D. (2005). Angew. Chem., Int. Ed..

[cit13] Zappa F., Denifl S., Mahr I., Lecointre J., Rondino F., Echt O., Mark T. D., Scheier P. (2007). Eur. Phys. J. D.

[cit14] Ptasinska S., Denifl S., Grill V., Mark T. D., Illenberger E., Scheier P. (2005). Phys. Rev. Lett..

[cit15] Abdoul-Carime H., Gohlke S., Illenberger E. (2004). Phys. Rev. Lett..

[cit16] Sanche L. (2002). Mass Spectrom. Rev..

[cit17] Kopyra J., Koenig-Lehmann C., Illenberger E. (2009). Int. J. Mass Spectrom..

[cit18] Ptasinska S., Denifl S., Mroz B., Probst M., Grill V., Illenberger E., Scheier P., Mark T. D. (2005). J. Chem. Phys..

[cit19] Profeta L. T. M., Larkin J. D., Schaefer H. F. (2003). Mol. Phys..

[cit20] Jiao D. S., Wang H. Y. (2008). Mol. Phys..

[cit21] Denifl S., Ptasinska S., Probst M., Hrusak J., Scheier P., Mark T. D. (2004). J. Phys. Chem. A.

[cit22] Chen E. C. M., Wiley J. R., Chen E. S. (2008). Nucleosides, Nucleotides Nucleic Acids.

[cit23] Parsons B. F., Sheehan S. M., Yen T. A., Neumark D. M., Wehres N., Weinkauf R. (2007). Phys. Chem. Chem. Phys..

[cit24] Wightman A. S. (1950). Phys. Rev..

[cit25] Wallis R. F., Herman R., Milnes H. W. (1960). J. Mol. Spectrosc..

[cit26] Garrett W. R. (1970). Chem. Phys. Lett..

[cit27] Garrett W. R. (1971). Phys. Rev. A.

[cit28] Stockdal J., Davis F. J., Compton R. N., Klots C. E. (1974). J. Chem. Phys..

[cit29] Haberland H., Ludewigt C., Schindler H. G., Worsnop D. R. (1984). J. Chem. Phys..

[cit30] Desfrancois C., Baillon B., Schermann J. P., Arnold S. T., Hendricks J. H., Bowen K. H. (1994). Phys. Rev. Lett..

[cit31] Hendricks J. H., Lyapustina S. A., deClercq H. L., Snodgrass J. T., Bowen K. H. (1996). J. Chem. Phys..

[cit32] Desfrancois C., Periquet V., Bouteiller Y., Schermann J. P. (1998). J. Phys. Chem. A.

[cit33] Hammer N. I., Diri K., Jordan K. D., Desfrancois C., Compton R. N. (2003). J. Chem. Phys..

[cit34] Hammer N. I., Hinde R. J., Compton R. N., Diri K., Jordan K. D., Radisic D., Stokes S. T., Bowen K. H. (2004). J. Chem. Phys..

[cit35] Zimmerman A. H., Brauman J. I. (1977). J. Chem. Phys..

[cit36] Wetmore R. W., Schaefer H. F., Hiberty P. C., Brauman J. I. (1980). J. Am. Chem. Soc..

[cit37] Jackson R. L., Hiberty P. C., Brauman J. I. (1981). J. Chem. Phys..

[cit38] Walthall D. A., Karty J. M., Romer B., Ursini O., Brauman J. I. (2005). J. Phys. Chem. A.

[cit39] Lykke K. R., Mead R. D., Lineberger W. C. (1984). Phys. Rev. Lett..

[cit40] Mead R. D., Lykke K. R., Lineberger W. C., Marks J., Brauman J. I. (1984). J. Chem. Phys..

[cit41] Lykke K. R., Neumark D. M., Andersen T., Trapa V. J., Lineberger W. C. (1987). J. Chem. Phys..

[cit42] Marks J., Brauman J. I., Mead R. D., Lykke K. R., Lineberger W. C. (1988). J. Chem. Phys..

[cit43] Lykke K. R., Murray K. K., Neumark D. M., Lineberger W. C. (1988). Philos. Trans. R. Soc., A.

[cit44] Wetzel D. M., Brauman J. I. (1989). J. Chem. Phys..

[cit45] Yokoyama K., Leach G. W., Kim J. B., Lineberger W. C. (1996). J. Chem. Phys..

[cit46] Yokoyama K., Leach G. W., Kim J. B., Lineberger W. C., Boldyrev A. I., Gutowski M. (1996). J. Chem. Phys..

[cit47] Liu H. T., Ning C. G., Huang D. L., Dau P. D., Wang L. S. (2013). Angew. Chem., Int. Ed..

[cit48] Simons J. (1981). J. Am. Chem. Soc..

[cit49] Berry R. S. (1966). J. Chem. Phys..

[cit50] Liu H. T., Ning C. G., Huang D. L., Wang L. S. (2014). Angew. Chem., Int. Ed..

[cit51] Desfrançois C., Abdoul-Carime H., Schermann J. P. (1996). J. Chem. Phys..

[cit52] Schiedt J., Weinkauf R., Neumark D. M., Schlag E. W. (1998). Chem. Phys..

[cit53] Leon I., Yang Z., Liu H. T., Wang L. S. (2014). Rev. Sci. Instrum..

[cit54] Wang L. S., Ding C. F., Wang X. B., Barlow S. E. (1999). Rev. Sci. Instrum..

[cit55] Wang X. B., Wang L. S. (2008). Rev. Sci. Instrum..

[cit56] Wang X. B., Woo H. K., Wang L. S. (2005). J. Chem. Phys..

[cit57] Liu H. T., Wang Y. L., Xiong X. G., Dau P. D., Piazza Z. A., Huang D. L., Xu C. Q., Li J., Wang L. S. (2012). Chem. Sci..

[cit58] Dau P. D., Su J., Liu H. T., Huang D. L., Li J., Wang L. S. (2012). J. Chem. Phys..

[cit59] Neumark D. M. (2008). J. Phys. Chem. A.

[cit60] Grumbling E. R., Sanov A. (2011). J. Chem. Phys..

[cit61] Dau P. D., Liu H. T., Huang D. L., Wang L. S. (2012). J. Chem. Phys..

[cit62] Garcia G. A., Nahon L., Powis I. (2004). Rev. Sci. Instrum..

[cit63] Dribinski V., Ossadtchi A., Mandelshtam V. A., Reisler H. (2002). Rev. Sci. Instrum..

[cit64] Western C. M., PGOPHER, a Program for Simulating Rotational Structure, University of Bristol, 2013, http://pgopher.chm.bris.ac.uk.

[cit65] Huang D. L., Zhu G. Z., Wang L. S. (2015). J. Chem. Phys..

[cit66] Fano U. (1961). Phys. Rev..

[cit67] Kim J. B., Weichman M. L., Yacovitch T. I., Shih C., Neumark D. M. (2013). J. Chem. Phys..

[cit68] Huang D. L., Dau P. D., Liu H. T., Wang L. S. (2014). J. Chem. Phys..

