# Supplementary material for: Probing the vibrational spectroscopy of the deprotonated thymine radical by photodetachment and state-selective autodetachment photoelectron spectroscopy via dipole-bound states
Source: Chem Sci. 2015 Mar 17;6(5):3129–38. doi: 10.1039/c5sc00704f (PMC5657408; doi:10.1039/c5sc00704f)
Supplement: Supplementary file 1 [file SC-006-C5SC00704F-s001.pdf]

Electronic Supplementary Material (ESI) for Chemical Science.
This journal is © The Royal Society of Chemistry 2015

Supplementary Information

Probing the Vibrational Spectroscopy of the Deprotonated Thymine Radical by Photodetachment and State-Selective Autodetachment Photoelectron Spectroscopy via Dipole-Bound States

Dao-Ling Huang,[1] Hong-Tao Liu,[1,2] Chuan-Gang Ning,[3] Guo-Zhu Zhu,[1] and Lai-Sheng Wang[1,a)]

[1]*Department of Chemistry, Brown University, Providence, Rhode Island 02912, USA*

[2]*Shanghai Institute of Applied Physics, Chinese Academy of Sciences, Shanghai 201800, China*

[3]*Department of Physics, State Key Laboratory of Low-Dimensional Quantum Physics, Tsinghua University, Beijing 100084, China*

[a)]Email: Lai-Sheng_Wang@brown.edu

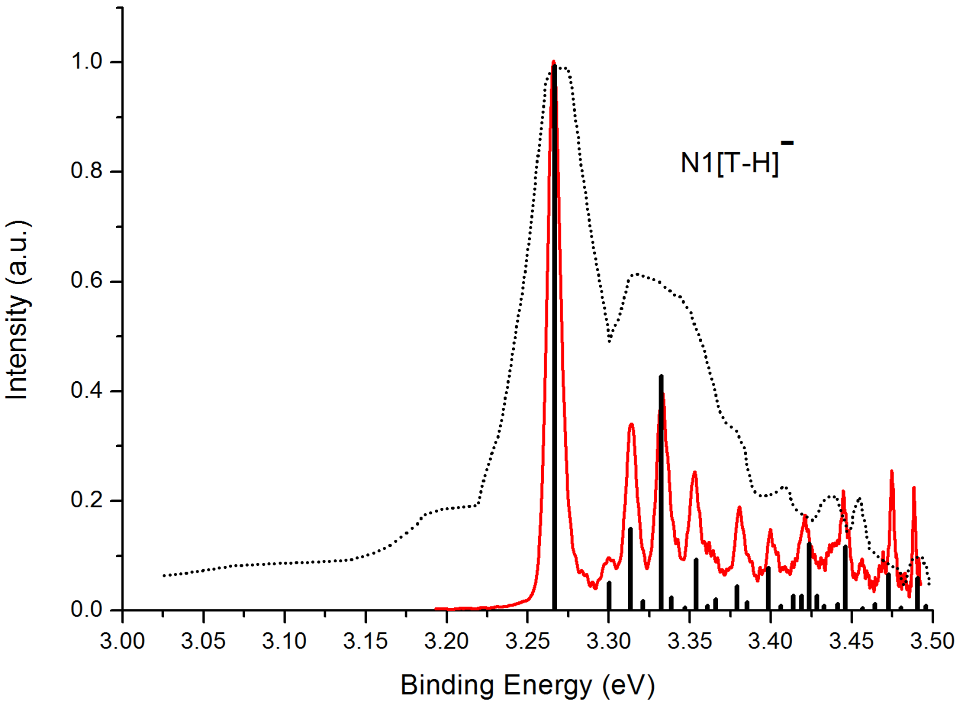

Figure S1. Comparison of the current 354.84 nm photoelectron spectrum (red) with the previous spectrum at the same wavelength (dotted line) and the previous Franck-Condon simulation (vertical bars) for the N1[T-H]$^-$ isomer.[23]

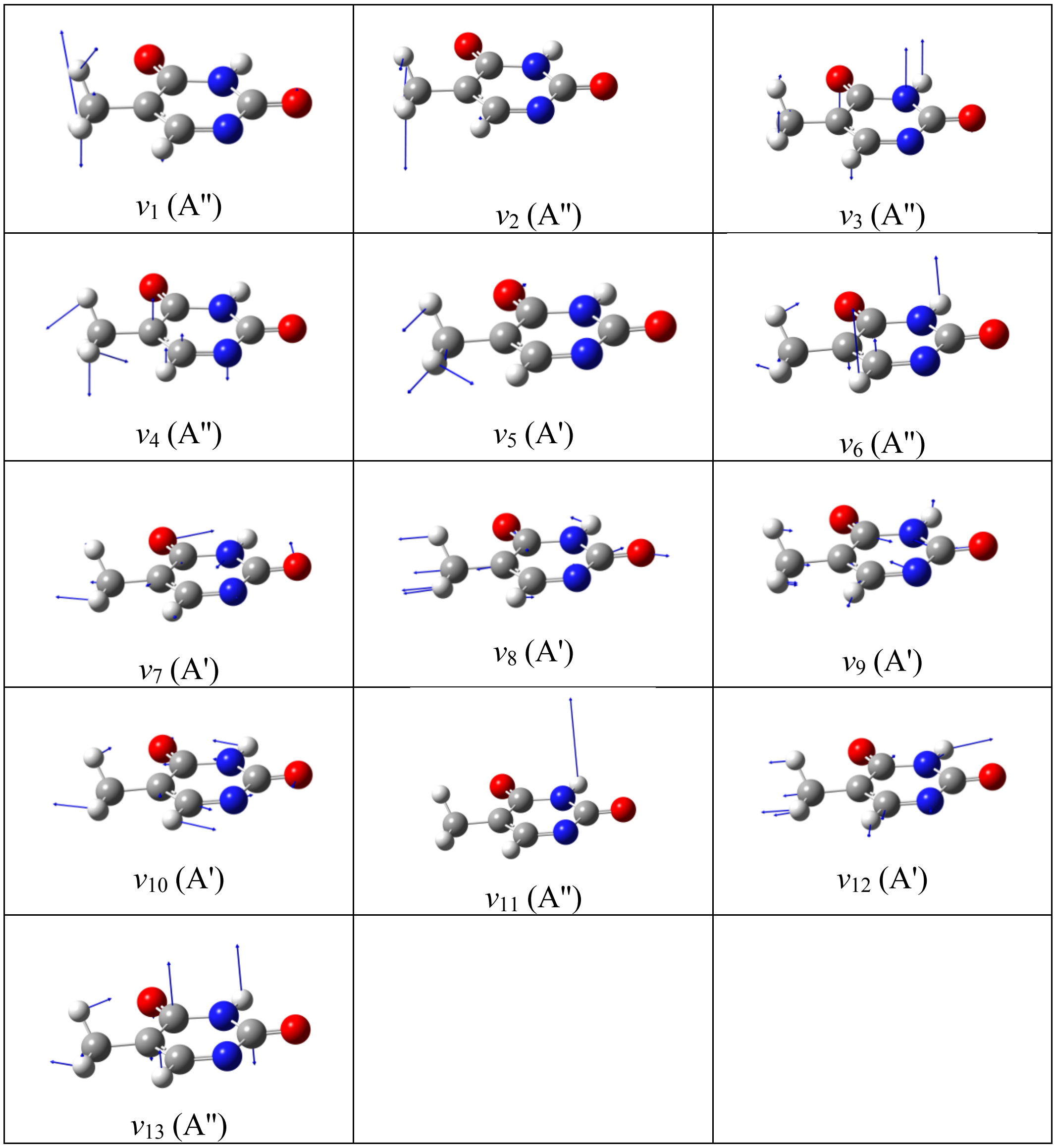

Figure S2. The thirteen lowest frequency normal modes of N1[T-H]•. The arrows indicate the displacement vectors. See Table S1 and Table 2 for their calculated frequencies.

Table S1 The complete list of the symmetries and vibrational frequencies of the N1[T-H]• radical calculated with the B3LYP/6-311++G(d, p) method.

Mode	Symmetry	Theoretical (cm^{-1})
v_1	A″	70
v_2	A″	93
v_3	A″	135
v_4	A″	262
v_5	A′	285
v_6	A″	388
v_7	A′	397
v_8	A′	450
v_9	A′	555
v_{10}	A′	608
v_{11}	A″	670
v_{12}	A′	720
v_{13}	A″	727
v_{14}	A″	751
v_{15}	A′	795
v_{16}	A′	918
v_{17}	A″	941
v_{18}	A′	998
v_{19}	A″	1019
v_{20}	A′	1179
v_{21}	A′	1223
v_{22}	A′	1300
v_{23}	A′	1342
v_{24}	A′	1402
v_{25}	A′	1413
v_{26}	A′	1423
v_{27}	A″	1453
v_{28}	A′	1484
v_{29}	A′	1488
v_{30}	A′	1708
v_{31}	A′	1720
v_{32}	A′	3021
v_{33}	A″	3063
v_{34}	A′	3121
v_{35}	A′	3127
v_{36}	A′	3581
